# Biochemical, Catabolic, and PGP Activity of Microbial Communities and Bacterial Strains from the Root Zone of *Baccharis linearis* in a Mediterranean Mine Tailing

**DOI:** 10.3390/microorganisms11112639

**Published:** 2023-10-26

**Authors:** Humberto Aponte, Yoelvis Sulbaran-Bracho, Pedro Mondaca, Catalina Vidal, Rodrigo Pérez, Sebastián Meier, Pablo Cornejo, Claudia Rojas

**Affiliations:** 1 Laboratory of Soil Microbial Ecology and Biogeochemistry, Institute of Agri-Food, Animal and Environmental Sciences (ICA3), Universidad de O’Higgins, San Fernando 3070000, Chile; claudia.rojas@uoh.cl; 2 Centre of Systems Biology for Crop Protection (BioSaV), Institute of Agri-Food, Animal and Environmental Sciences (ICA3), Universidad de O’Higgins, San Fernando 3070000, Chile; 3 Laboratory of Entomology, Institute of Agri-Food, Animal and Environmental Sciences (ICA3), Universidad de O’Higgins, Rancagua 2841959, Chile; 4 Center of Biotechnology “Dr. Daniel Alkalay Lowitt”, Universidad Técnica Federico Santa María, General Bari 699, Valparaíso 2390136, Chile; 5 Departamento de Ciencias Químicas y Recursos Naturales, Facultad de Ingeniería y Ciencias, Campus Andrés Bello, Universidad de La Frontera, Avenida Francisco Salazar, Temuco 4811230, Chile; catalina.vidal@ufrontera.cl (C.V.); rodrigo.perez@ufrontera.cl (R.P.); 6 Doctorate Program in Sciences of Natural Resources, Universidad de la Frontera, Temuco 4811230, Chile; 7 Instituto de Investigaciones Agropecuarias (INIA), Centro de Investigación Regional de Investigación Carillanca, Temuco 4880815, Chile; sebastian.meier@inia.cl; 8 Escuela de Agronomía, Facultad de Ciencias, Ingeniería y Tecnología, Campus Alemania Sede Temuco, Universidad Mayor, Av. Alemania 0281, Temuco 4801043, Chile; 9 Escuela de Agronomía, Facultad de Ciencias Agronómicas y de los Alimentos, Pontificia Universidad Católica de Valparaíso, Quillota 2260000, Chile; pablo.cornejo@pucv.cl; 10 Center of Applied Ecology and Sustainability (CAPES), Santiago 8331150, Chile

**Keywords:** rhizosphere, mining waste, community-level physiological profile, plant growth promoting

## Abstract

The management of mine tailings (MT) is commonly workload heavy, intrusive, and expensive. Phytostabilization offers a promising approach for MT management; however, it poses challenges due to the unfavorable physicochemical properties of these wastes. Nevertheless, native microorganisms capable of supporting plant growth and development could enhance the efficacy of phytostabilization. This study assesses the biological activity of microbial communities from the root zone of *Baccharis linearis*, which is naturally present in MT, in order to evaluate their biotechnological potential for phytostabilization. The root zone and bulk samples were collected from *B. linearis* plants located within a MT in the Mediterranean zone of Chile. Enzyme activities related to the cycling of C, N, and P were assessed. The community-level physiological profile was evaluated using the MicroResp^TM^ system. Bacterial plant growth-promoting (PGP) traits and colony forming units (CFU) were evaluated through qualitative and microbiological methods, respectively. CFU, enzyme activities, and CLPP were higher in the root zone compared with the bulk samples. Five bacterial strains from the root zone exhibited PGP traits such as P solubilization and N acquisition, among others. The presence of microbial communities in the root zone of *B. linearis* with PGP traits suggests their potential to enhance the ecological management of MT through phytostabilization programs.

## 1. Introduction

Mine tailings are the byproducts of mineral ore mining [1], primarily distinguished by their elevated metal and metalloid contents [hereafter referred to as metal(loid)s], limited nutrient availability, acidic pH, and inadequate physical structure, among other characteristics [2]. Consequently, the disposal of mine tailings typically leads to detrimental impacts on ecosystem functioning, manifesting as adverse effects on both plant life and soil microorganisms [1,3]. This is the case with on-land tailing disposal, a practice that not only occupies extensive land areas [4] but also generates negative repercussions for ecosystems due to the following factors: (1) high metal(loid) content, which can negatively affect soil biological activity and its mediated biogeochemical cycling [5]; (2) the presence of fine materials, which can contaminate soil, water, and air, thereby affecting both neighboring regions and human populations [2]; (3) poor physical properties, creating a susceptibility to potential collapses and landslides of deposits, particularly during rainy seasons [6]; (4) the potentially high content of residuals agents, such as sulfides, which can promote acidification processes that increase metal(loid) mobilization and toxicity [2].

Various techniques have been explored to mitigate the negative effects of mine tailings; however, physical and chemical approaches are constrained by limitations in terms of effectiveness, labor intensity, and economic investment [7]. In this context, biological treatments, including phytoremediation, have emerged as sustainable solutions for the management of mine tailings due to their cost effectiveness and non-invasive nature [8]. However, in Mediterranean regions, phytoremediation of mine tailings can pose challenges and prove ineffective due to the unfavorable physicochemical properties of these materials, limited water availability, and a restricted range of plant species capable of thriving under such adverse conditions, thereby avoiding the inadvertent phytoextraction of metal(loid)s [9]. Therefore, phytostabilization should be complemented with other biotechnological tools, such as incorporating organic amendments and microorganisms, to enhance the efficacy of phytostabilization in mine tailings [9,10].

Numerous studies have reported the presence of microbial groups in mine tailings, particularly in the rhizosphere of different plant species, which play roles in essential ecological processes [11]. Several studies have found a prevalence of Proteobacteria, Acidobacteria, Chloroflexi, Actinobacteria, and other taxa in various mine tailings, exhibiting advantageous traits for plant growth and development [11,12,13]. In this context, microbial communities act as pioneer colonizers, playing pivotal roles in elemental transformation, carbon and nitrogen fixation, and the enhancement of mineral soil fertility and plant growth [14]. For instance, Pérez et al. [15] reported higher shoot and root dry weights in *Oenothera piscencis* growing in mine tailings after inoculation with *Claroideoglomus claroideum*. Similarly, Gazitúa et al. [16] found greater plant height, number of leaves, secondary root counts, and dry biomass in both shoot and root when seeds of *Baccharis linearis* were sown in mine tailings compared to sterilized mine tailings. Conversely, Romero et al. [17] observed increased survival and development of *Populus nigra* by inoculation with a consortium of bacterial strains exhibiting PGP traits, isolated from a mine tailing. Furthermore, these authors did not observe the effects of inoculation with a commercial mycorrhizal inoculum. Hence, microorganisms linked with native plants capable of thriving in mine tailings hold significant interest, especially considering that native plant species are currently favored in phytoremediation programs [18,19]. Nevertheless, there are a lack of studies focused on PGP microorganisms derived from mine tailings, necessitating further investigation into such microorganisms [9,20].

Given the relevance of native plant species, *B. linearis* emerges as a promising candidate for phytoremediation programs. *B. linearis* (Asteraceae) is a deciduous evergreen shrub present in north-central Chile, which displays adaptation to degraded soils and Mediterranean semiarid environments [21]. This species has been recognized as a spontaneous colonizing species in abandoned mine tailings [22], with rhizosphere microbial communities able to improve plant acclimation in mine tailings [16]. The rhizosphere of *B. linearis* hosts a diverse range of bacterial assemblages, including Actinobacteria, Gammaproteobacteria, and Firmicutes [16]. These constituents demonstrate distinct metabolic capabilities and adaptive resistance to diverse environmental conditions within their respective taxa [23,24]. Additionally, *B. linearis* has been described as a nurse species that facilitates secondary colonization [25], potentially driving ecological succession. Although microbial communities inhabiting the rhizosphere of *B. linearis* have been described in few studies [16,26], there has been no evaluation of their functionality and their potential plant growth-promoting (PGP) traits. Therefore, this study aims to assess the biological activity of microbial communities from both the root zone and bulk soil (i.e., bulk samples of mine tailings) associated with naturally occurring *B. linearis* in a Cu abandoned mine tailing, to assess their potential as biotechnological tools for phytostabilization programs.

## 2. Materials and Methods

### 2.1. Study Site

**Figure 1:**
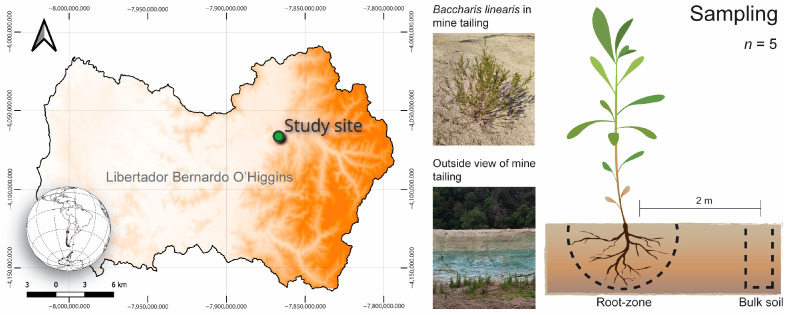
Study site and sampling scheme for the root zone and bulk soil. The “outside view of mine tailing” shows the tailing deposit (at the top) and the creek at the bottom, which appears dry in the picture.

The study site was located within “El Teniente” mine (CODELCO) in the Libertador Bernardo O’Higgins Region, Chile (34°16′44.87” S, 70°40′10.01” W) (Figure 1), which is the major Cu and Mo mine producer in Chile. The El Teniente CODELCO division accumulates the largest Cu waste deposit worldwide [11] and is located within the Mediterranean climate zone of central Chile. This region is characterized by an average annual rainfall of 28.6 mm [27], an average annual temperature of 13.7 °C, and average annual relative humidity of 42.7% [28]. During summer, temperatures can soar up to ~32 °C without any accompanying precipitation [28]. The selected tailing area consists of a relatively level terrain, surrounded by native Mediterranean sclerophyll forests on steep slopes toward the north-east, and by a minor tributary creek flowing to the Cachapoal River and towards the south-east (Figure 1). While the study site is enveloped by native sclerophyll forest, only a few native species, such as *B. linearis* and *Eschscholzia californica*, were found within the mine tailings. Among these, *B. linearis* emerged as the most abundant and enduring under field conditions. Thus, within the flat tailing area, a patch of *B. linearis* in a small tailing mixed with soil transported from uphill was chosen for this study.

Five plants of *B. linearis* were selected based on their height (50–60 cm) and the absence of associations with other plant species. This was performed to avoid deep and large woody roots. For each plant, two sampling areas were delimited (Figure 1): (1) the root zone (soil around roots, at a depth of 5–20 cm, and within a radius of 20 cm from the stem); (2) bulk soil areas situated two meters away from the plants, in sections without any plant cover (at a depth of 5–20 cm). For the root-zone samples, the plant was carefully pulled off until the root system was exposed; the soil adhering to the roots and the soil in close proximity were collected aseptically to form a composite sample per plant. For the bulk soil samples, three random points distanced two meters away from the plant were considered to create the composite sample. Each composite sample (~1 kg) was constituted by combining four subsamples of ~250 g each. The collected samples were promptly refrigerated in the field and subsequently transported to the laboratory for further analysis. In the laboratory, samples were sieved (2 mm mesh), homogenized, stored at 4 °C for two weeks, and air dried for physicochemical analysis.

### 2.2. Physicochemical Characterization of Mine Tailings

Soil pH and electrical conductivity (CE) were determined in a soil–deionized water extract (at a ratio of 1:2.5 and 1:5 *w*/*v*, respectively), following the method by Sadzawka et al. [29]. Total nitrogen content was quantified using Kjeldahl digestion [30]. The available P (AP) was determined by the Olsen method (extraction with 0.5 M NaHCO_3_ at pH 8.5), followed by analysis via the blue molybdate method [31,32]. Soluble contents of Ca, Mg, Cu, and Zn were determined in the saturated paste extract to reflect the field conditions of the experiment and minimize the impact of substrate granulometry [33]. Concentrations of these elements in the saturated paste extract were determined using flame atomic absorption spectroscopy (FAAS, iCE 3300, Thermo Fisher, Waltham, MA, USA), with lanthanum added for Ca and Mg measurements. Total concentrations of other nutrients and trace elements (P, K, S, Fe, As, Mn, and Mo) were estimated using a portable X-ray fluorescence spectrometer (XRF, S1 Titan Bruker, Berlin, Germany, with GeoChem method). For this analysis, soil samples were examined in triplicate following the US EPA protocol method 6200 for sample processing [34]. Quality was assured by analyzing ISE-973 and ISE-859 certified reference samples (Wageningen University, Netherlands). The results obtained for these standard reference materials were found to be within 10% of the certified values (except for As, where the difference was 20%). Quality control was maintained by employing standards and blanks.

### 2.3. Soil Enzyme Activities

Enzyme activities determined include the following: (1) fluorescein diacetate hydrolysis (FDA) by the fluorescein released after soil incubation with FDA [35]; (2) dehydrogenase activity (DHA) by the reduction in INT [2-(*p*-iodophenyl)-3-(*p*-nitrophenyl)-5-phenyltetrazolium chloride] to INTF (iodonitrotetrazolium formazan) [36]; (3) β-glucosidase activity (Gluc) by the *p*-nitrophenol released after soil incubation using *p*-nitrophenyl-β-D-glucopyranoside as the substrate [37]; (4) arginine ammonification (AA) by the measurement of ammonium released after soil incubation with arginine [38]; (5) urease activity (UA) by the ammonium determination after soil incubation with urea [39]; (6) acid phosphatase activity (Pacid) by the release of *p*-nitrophenol after soil incubation with *p*-nitrophenyl-phosphate as substrate [40]. The enzyme activities were measured with a microplate spectrophotometer (Epoch2, BioTek, Winooski, VT, USA). Pacid, DHA, and FDA were assessed in UV-V cuvettes to avoid reading errors due to volatilization and the presence of precipitates.

### 2.4. Community-Level Physiological Profile

The community-level physiological profile (CLPP) was determined with the MicroResp^TM^ system (MicroResp^TM^, James Hutton Ltd., Aberdeen, UK) according to Chapman et al. [41]. The C substrate used were the following: (1) carbohydrates: glucose (GLU), fructose (FRU), L-arabinose (ARA), N-acetyl glucosamine (NAGA), trehalose (TRE); (2) carboxylic acids: γ-aminobutyric acid (GABA), α-ketoglutaric acid (AKGA), oxalic acid (OXA), citric acid (CIT), malic acid (MAL); (3) amino acids: L-arginine (ARG), cysteine (CYS), lysine (LYS); (4) phenolic acids: protocatechuic acid (PRO); (5) polymeric: α-cyclodextrin (ACYC). Additionally, an incubation with water (WAT) was included. The microbial CO_2_ emission was estimated after 6 h of incubation at 25 °C. For each sample, the CO_2_ emission resulting from the utilization of each C-source was averaged to determine microbial catabolic activity, quantified as the average well color development (AWCD) [42]. The colorimetric measurement was conducted using a microplate spectrophotometer (Epoch2, BioTek, Winooski, VT, USA).

### 2.5. Bacterial Plant Growth-Promoting Traits

The quantification of culturable aerobic heterotrophic microorganisms’ colony forming units (CFUs) was determined according to Maier et al. [43]. The cultivable microorganisms from the root zone were isolated using serial dilutions. Specifically, 1 g of soil sample was taken and suspended in 9 mL of sodium chloride solution (0.9% *w*/*v*). The resulting dilutions were plated on Luria-Bertani (LB) agar and incubated at 30 °C for 2–3 days, with daily monitoring until the formation of microbial colonies on the agar surface was observed. Bacterial strains were chosen based on their morphological characteristics, such as color, elevation, and edge. This selection method follows the approach established by Leontidou et al. [44], Majeed et al. [45], and Patel et al. [46]. Five colonies representing each observed morphology were selected, taking into account the possibility of distinct strains within the same bacterial species that exhibit similar morphologies. In total, 48 bacterial strains with distinct morphologies were selected.

The plant growth-promoting (PGP) traits of the selected strains were evaluated through pure isolation using the following methods: (1) nitrogen fixation by the incubation (30 °C by 7 days) in agar (1.5% *v*/*v*) with nitrogen-fixing bacteria medium (NFB) [47]; (2) phosphate solubilization by the incubation (30 °C by 7 days) in Pikovskaya’s medium with bromophenol blue (0.16% *v*/*v*) and LB agar (1.5% *v*/*v*); (3) the activity of ethylene 1-amino-cyclopropane carboxylic acid (ACC) deaminase by incubation in plates with Dworkin and Foster (DF) medium [48] and ACC (3 mM) by 48 h at 30 °C; (4) production of indole acetic acid (IAA) by microbial growing in LB/YPF agar with tryptophane (0.1% *v*/*v*) by five days at 30 °C, from which 1 mL is treated with 1 mL of the Salkowski solution and incubated (30 °C) for further colorimetric reading at 536 nm; (5) production of siderophores by using blue agar medium (CAS) according to [49], where isolates were incubated in CAS agar at 30 °C for five days. The strains that clearly exhibited at least two out of the five positive tests for in vitro PGP traits were selected.

For the extraction of isolate DNA, the strains were cultivated in LB medium for 24 h in an orbital shaker at 180 rpm and a temperature of 30 °C. Subsequently, they were centrifuged at 8300 rpm and washed with a NaCl solution (0.9% *w*/*v*) to remove the culture medium. This washing step was repeated three times. The DNA from each sample was purified using the “Wizard Genomic DNA Extraction” kit from PROMEGA. The integrity and concentration of the DNA were assessed using 1% agarose gels and the spectroscopic ratios of OD_260_/_280_, respectively. The 16S rRNA gene was amplified using the universal primers 1492R and 27F [50], and the amplification products were then sequenced at the Centre for Automatic DNA Sequencing, Pontificia Universidad Católica de Chile. The resulting sequences were edited and assembled using the BIOEDIT v7.2 [51] software, and analyzed using the Basic Local Alignments Search Tool (BLAST) program available from the National Centre for Biotechnology Information (NCBI), to determine the identity of the sequences obtained.

### 2.6. Phylogenetic Analysis

The 16S rRNA gene sequences from isolates were aligned using MAFFT v7 [52], and reference sequence fragments of the 16S rRNA gene were obtained from nearby organisms through NCBI. The phylogeny of the aligned sequences was inferred with FastTree v2.1.11 [53] with a generalized time-reversible model.

### 2.7. Statistical Analyses

Results were analyzed considering the sample source (root zone and bulk soil) as a fixed factor. The analyses were performed in R Statistics version 4.2.2 [54]. Normality and homoscedasticity assumptions were assessed using the Shapiro–Wilk and Levene tests, respectively. A Student *t*-test was applied for all variables that met statistical assumptions. In cases where experimental variables did not meet statistical assumptions, a log transformation was applied; the same transformation was also performed for CFUs. A Pearson correlation test was applied between all variables with the functions “cor” and “corrplot” from the package “corrplot” [55]. The CLPP was analyzed using a principal coordinate analysis (PCoA) and PERMANOVA with the package “vegan” [56]. Additionally, a heatmap cluster analysis was performed on the C substrate utilization from the CLPP with the “heatmap” function. Furthermore, a principal component analysis (PCA) was conducted using the “FactoMineR” [57] and “factoextra” [58] packages for multivariate evaluation. This PCA considered sampling points based on physicochemical and biological properties, and grouping ellipsoids were assessed through a PERMANOVA based on Euclidean distance.

## 3. Results

### 3.1. Physicochemical Properties

**Figure 2:**
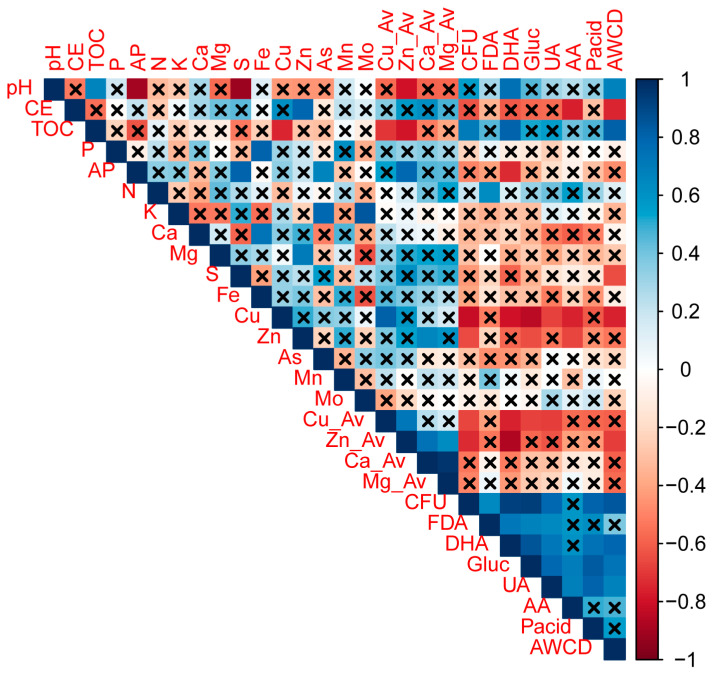
Spearman correlation matrix between physicochemical and biological properties. Crossed boxes represent non-significant correlations (*p* > 0.05). Blue boxes show positive correlations, while red boxes show negative correlations. CE = electrical conductivity; TOC = total organic carbon; AP = available P; CFU = colony forming units; FDA = fluorescein diacetate hydrolysis; DHA = dehydrogenase activity; Gluc = β-glucosidase activity; UA = urease activity; AA = arginine ammonification; Pacid = acid phosphatase; AWCD = average well color development.

Physicochemical properties of the root zone did not significantly vary compared to those of the bulk soil samples, except for the total contents of Fe and Cu, as well as available Zn, which were lower in the root-associated zone than in the bulk soil (Table 1). However, although non-significant differences were observed, the pH showed variation from very strongly acidic (4.77 ± 0.28) in the bulk soil to strongly acidic (5.30 ± 0.52) in the root zone. Similarly, TOC was slightly higher in the root-associated zone compared to the bulk soil, while CE exhibited a lower trend in the root zone (Table 1). Additionally, the available contents of Cu and Zn (Cu_H2O_ and Zn_H2O_) were lower in the root zone compared to the bulk soil, showing negative correlations with pH (r = −0.65 and −0.79, respectively; see Figure 2).

### 3.2. Enzyme Activities, CFUs, and CLPP

**Figure 3:**
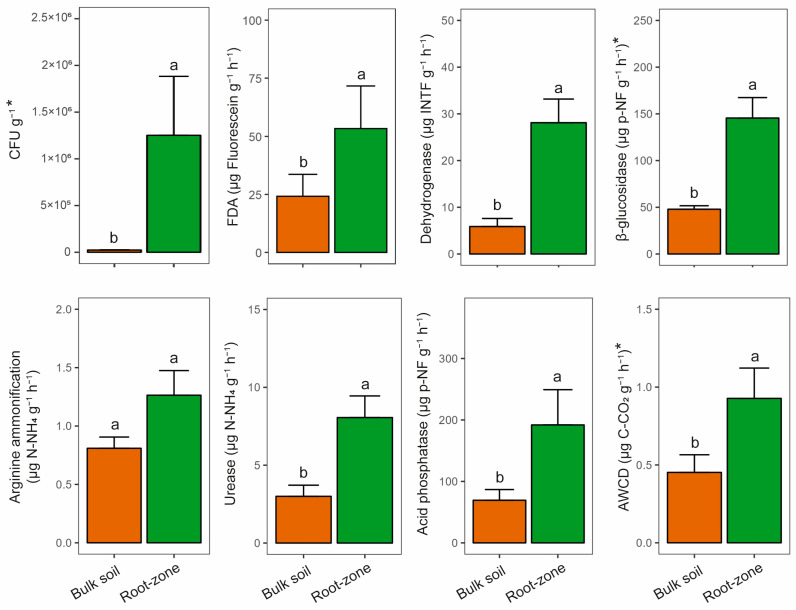
Colony forming units (CFU), enzyme activities, and catabolic capacity of microbial communities in the root zone and bulk soil (*n* = 5, mean ± s.e.). FDA = fluorescein diacetate hydrolysis (FDA), AWCD = average well color development. Different letters (“a” and “b”) show significant differences according to the Student *t*-test (*p* ≤ 0.05). * = Log-transformed variables.

**Figure 4:**
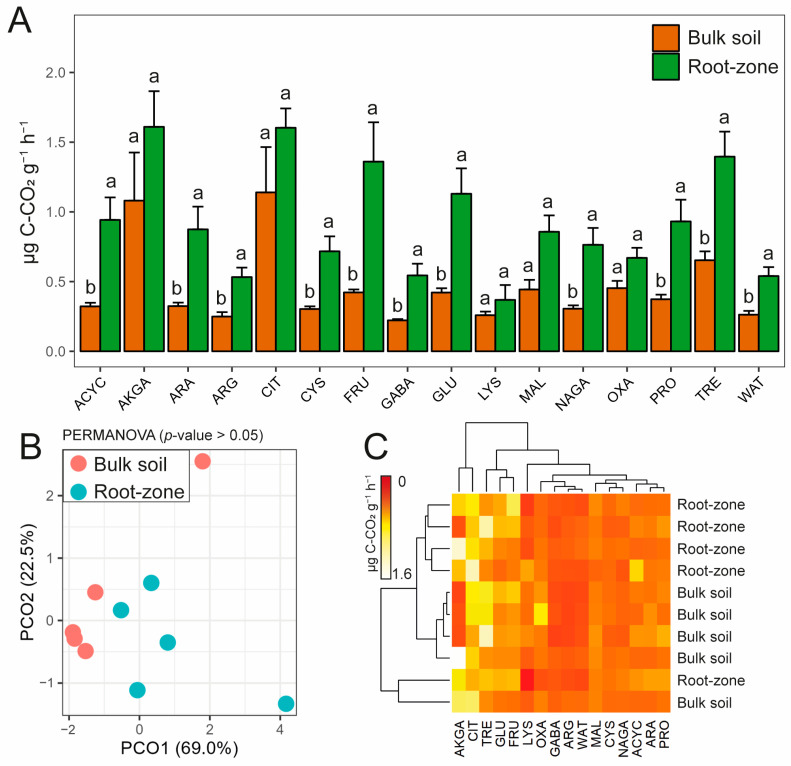
Community-level physiological profile (CLPP) by (**A**) individual carbon source utilization (*n* = 5; mean ± s.e.), different letters show significant differences (*p* ≤ 0.05) according to a Student *t*-test; (**B**) principal coordinate analysis and PERMANOVA based on Euclidean distances; (**C**) heatmap scaled by each carbon source utilization. ACYC = α-cyclodextrin, AKGA = α-ketoglutaric acid, ARA = L-arabinose, ARG = L-arginine, CIT = citric acid, FRU = fructose, GABA = γ-amino butyric acid, GLU = glucose, CYS = cysteine, LYS = lysine, MAL = malic acid, NAGA = N-acetyl glucosamine, OXA = oxalic acid, PRO = protocatechuic acid, TRE = trehalose, WAT = water.

Most of the biological properties were higher in the root zone compared to those in the bulk soil (Figure 3 and Figure 4A). CFUs were significantly higher (50 times greater) in the root zone than in the bulk soil (Figure 3), exhibiting a positive correlation with TOC (r = 0.81) and negative correlations with the total and available Cu and Zn contents (Figure 2). Similarly, all enzyme activities were higher in the root zone compared to the bulk soil, except for AA. DHA was over three times higher in the root zone compared to the bulk soil (root zone = 28.10 ± 11.33; bulk soil = 5.88 ± 3.85 µg INTF g^−1^ h^−1^), followed by Gluc (root zone = 145.43 ± 48.93; bulk soil = 47.90 ± 8.37 µg p-nitrophenol g^−1^ h^−1^), Pacid (root zone = 191.92 ± 57.33; bulk soil = 69.36 ± 17.84 µg p-nitrophenol g^−1^ h^−1^), UA (root zone = 8.06 ± 3.11; bulk soil = 3.00 ± 1.6 µg N-NH4 g^−1^ h^−1^), and FDA (root zone = 52.38 ± 18.42; bulk soil = 24.16 ± 9.43 µg Fluorescein g^−1^ h^−1^). FDA, DHA, and Gluc displayed negative correlations with the total and available Cu content (Figure 2). Additionally, DHA exhibited negative correlations with CuH_2_O and ZnH_2_O (r = −0.65 and −0.74, respectively).

The average catabolic activity, as assessed by the AWCD obtained from the CLPP, doubled in the root zone compared to that in the bulk soil (Figure 2), exhibiting positive correlations with pH and TOC (r = 0.84 and 0.82, respectively), and negative correlations with Zn_H2O_ (−0.67, respectively; Figure 3). Similar to enzyme activities, most carbon sources were utilized more in the root zone than in the bulk soil, except for carboxylic acids such as AKGA, CIT, MAL, OXA, and LYS, which showed the same trend without significant differences (Figure 4A). The FRU was the substrate most used in the root zone compared to the bulk soil (twice as much), followed by ACYC, ARA, GLU, and NAGA. Despite this, no significant differences were observed in the PCoA and PERMANOVA results (Figure 4B). Carbon substrate utilization was grouped into four clusters based on their overall higher usage in the CLPP (Figure 4C): (1) AKGA and CIT > (2) TRE, GLU, and FRU > (3) LYS, OXA, GABA, ARG, WAT > (4) MAL, CYS, NAGA, ACYC, ARA, and PRO.

### 3.3. Multivariate Analysis

**Figure 5:**
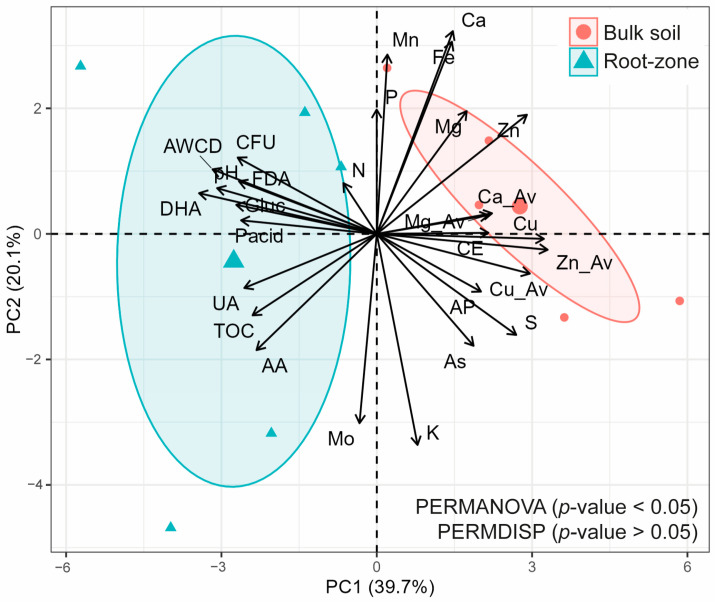
Principal component analysis (PCA) between physicochemical and biological properties and PERMANOVA and PERMDISP. CE = electrical conductivity; TOC = total organic carbon; AP = available P; CFU = colony forming units; FDA = fluorescein diacetate hydrolysis; DHA = dehydrogenase activity; Gluc = β-glucosidase activity; UA = urease activity; AA = arginine ammonification; Pacid = acid phosphatase; AWCD = average well color development.

Principal component analysis (PCA) and PERMANOVA of physicochemical and biological properties showed clear multivariate differences between the root zone and bulk soil (Figure 5), which was not evidenced with CLPP (Figure 4B). Additionally, PCA showed a higher and positive contribution of pH and biological properties (except for AA) in the root zone, while available nutrient and metal contents showed similar but negative trends in the bulk soil (Figure 5). This related to higher biological activity in the root zone, where nutrient and metal contents were slightly lower than in the bulk soil. In this context, PC1 and PC2 explained 40.8% and 19.8% of total variability, which accounted for a total of 60.6% based on both components (Figure 5).

### 3.4. Plant Growth-Promoting Traits

**Figure 6:**
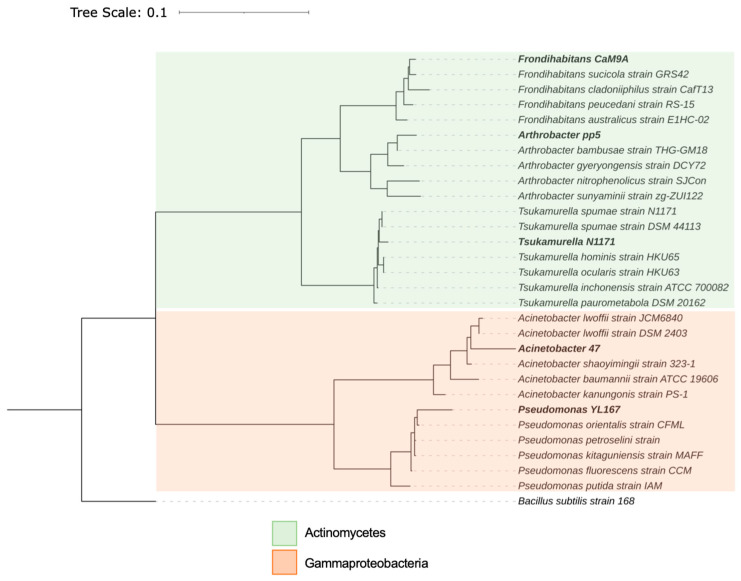
Phylogenetic Tree based on 16S rRNA gene sequences of phylotypes isolated from the root zone of *Baccharis linearis* in mine tailings. The tree was produced using the maximum likelihood with the general time reversible (GTR) model and rooted with *Bacillus subtilis* strain 168. The scale presented represents the changes per nucleotide position.

Five bacterial strains with different morphologies and PGP traits were isolated by evaluating the obtained colonies. Analysis of the 16S rRNA gene sequence revealed that isolates are classified within the class Actinomycetes and Gammaproteobacteria (Figure 6). In addition, the blast analysis in the GeneBank database showed that isolated bacterial strains belonged to the genera *Pseudomonas* (strain YL167), *Arthrobacter* (strain pp5), *Acinetobacter* (strain 47), *Frondihabitans* (strain CaM9A), and *Tsukamurella* (strain N1171). On the other hand, the isolates were phylogenetically related (i.e., clustered) with specific reference strains for each of them; the *Pseudomonas* YL167 strain was clustered in the phylogenetic tree with the *Pseudomonas orientalis* strain. In the same way, the *Arthrobacter* pp5 strain with *Arthrobacter bambusae*, the *Frondihabitans* CaM9A strain with *Frondihabitans sucicola*, the *Acinetobacter* 47 strain with *Acinetobacter iwoffi*, and the *Tsukamurella* N1171 strain with *Tsukamurella spumae* (Figure 6).

All the isolated bacteria showed activity of 1-aminocyclopropane-1-carboxylate deaminase activity (ACC), while only *Pseudomonas* sp. YL167, *Arthrobacter* sp. pp5, and *Tsukamurella* sp. N1171 showed the ability to fix nitrogen (Table 2) and are the bacterial strains that showed the qualitative greatest number of PGP traits (Table 2). On the other hand, compatibility tests can be performed, from which a consortium can be performed with all bacterial isolates. For this, we recommend the evaluation of all bacterial strains in a consortium to promote functional diversity and redundancy when applied in soils.

## 4. Discussion

The Cu content and pH in the bulk samples were similar to those reported by Ginocchio et al. [19] for mine tailings in a closely located area within the same study area. The total contents of Fe and Cu, as well as the available Zn, were lower in the root zone compared to the bulk soil, which is similar to that reported in the literature when assessing Cu and Zn contents in the rhizosphere and bulk soil in mine tailings [12,13]. Contrarily, Ginocchio et al. [59] reported higher Cu content beneath *B. linearis* shrubs in metal(loid) contaminated soils, attributing it to the physical retention of metal-enriched particles by the plant leaves. However, in the current study, young *B. linearis* individuals were considered, and thus the aerial retention of particles might be limited. Furthermore, Ginocchio et al. [59] reported Cu interaction with the organic detritus of *B. linearis*. In this context, Cu^2+^ can form stable complexes with carboxylic, amine, and thiol groups of organic matter [60,61]. Thus, Cu could have been immobilized on the organic fraction of soil below the plant and washed down by superficial water and wind effect, which can be associated with negative correlations between the available Cu content and TOC (r = −0.71). Total Cu content was high in all samples considering a range of reference contents for mine tailings and soils [62].

Few studies have evaluated the rhizosphere of *B. linearis*, especially by assessing soil biological properties. Among these, Cornejo et al. [63] reported higher total glomalin-related soil protein (GRSP) in the rhizosphere of *B. linearis* compared to the bulk soil in a metal(loid) contaminated area, which occurred due to the presence of arbuscular mycorrhizal fungi. Additionally, Gazitúa et al. [16] identified bacteria (*Gammaproteobacteria*, *Firmicutes*, and *Actinobacteria*) and fungi (arbuscular mycorrhiza) associated with the root zone of *B. linearis* in a Cu mine tailing, which promoted the plant establishment and development in mine tailings according to experimental assays. Consequently, the rhizodeposition and organic matter incorporation by *B. linearis* could potentially promote microbial recruitment to the root zone, which results in higher CFUs and microbial activity [16] in comparison to the bulk soil.

The highest enzyme activity and AWCD in the root zone can be associated with a high microbial biomass and organic matter, as well as lower Cu content as shown by relationships between CFUs, enzyme activities (except for AA), and total and available Cu contents (Figure 3). The positive effect of organic matter (slightly higher in the root zone) on soil biological activity has been widely described [64] even in metal contaminated soils [65]. In this context, organic matter serves as a substrate for heterotrophic microorganisms [66] and facilitates the creation of enzyme-humus complexes that mitigate the proteolytic and thermal degradation of extracellular enzymes (e.g., Pacid and UA) [67]. Also, organic matter can increase soil pH (as found here), thereby decreasing the availability and toxicity of metal(loid)s [68]. Results suggest that C, P, and N cycling are promoted in the root zone since (1) DHA and Gluc are related to the oxidation of organic compounds and cellulose hydrolysis to glucose releasing, respectively [37]; (2) Pacid releases phosphate from organic matter [40,69]; (3) UA breaks the urea’s C-N bond [39]; (4) AWCD, obtained from CLPP, reflect the overall microbial catabolic activity associated with C cycle [70].

Multivariate analysis of CLPP did not reveal significant differences between the root zone and bulk soil. However, the microbial utilization of most C sources, particularly carbohydrates, was higher in the root zone, with the exception of carboxylic acids. Such results agree with previous studies that have reported stronger negative effects of soil metal contents on the microbial utilization of carbohydrates [65,70]. Consequently, catabolic activity is promoted in the root zone of *B. linearis*, which was expected considering the higher CFUs, the rhizodeposition in the root zone [16], and the “Cry for Help” phenomenon (i.e., the release of certain plant metabolites to recruit beneficial microbial communities) that commonly occur in extreme environments [71]. Therefore, different microbial community structure could be found in the root zone compared with bulk soil, which can be largely moderated by the availability and turnover of organic compounds.

Bacterial isolates, obtained from the root zone of *B. linearis*, were taxonomically classified into the genera *Pseudomona*, *Arthrobacter*, *Frondihabitans*, *Acinetobacter,* and *Tsukamurella*. In the same study area, Galvez et al. [11] reported a dominant presence (>95% of the total abundance) of the families Pseudomonadaceae, Flavobacteriaceae, and Erwiniaceae from the bulk soils, with the presence of similar genera as found in this study such as Pseudomonas and Arthrobacter, and order such as Micrococcales. Similarly, Gazitúa et al. [16] found dominance of phylum Proteobacteria, Firmicutes, and Actinobacteria in the rhizoplane and rhizosphere of *B. linearis* in a Cu mine tailing. The presence of *Proteobacteria* (e.g., *Pseudomonas* sp. YL167 and *Acinetobacter* sp. 47) was expected in the root zone of *B. linearis* since such phylum is commonly dominant in the studied mine tailing [16] and rhizosphere of many plant species [72].

From the isolated bacteria, *Pseudomonas* sp. YL167, *Tsukamurella* sp. N1171, and *Arthrobacter* sp. pp5 showed all of the PGP traits assessed here. *Pseudomonas orientalis*, clustered with *Pseudomonas* sp. YL167, has been shown to have a high tolerance to Cu stress, showing PGP traits such as phosphate solubilization, and production of siderophore, IAA, and organic acids, with final stimulation of plant growth [73]. Additionally, *P. orientalis* has been described as antagonistic against phytopathogens such as *Erwinia amylosora* [74], which belongs to the Erwiniaceae family, one of the main families of bacteria found in most of the bulk soil in the same study area [11]. The *Arthrobacter* genus is widely described for presenting resistance to adverse conditions, being able to sequester metals and promote plant growth under stressful conditions with high metal contents [75,76]. Furthermore, *Arthrobacter* is able to alleviate drought stress [77], which is an important abiotic factor in abandoned mine tailings. The *Tsukamurella* genus are saprophytes [78], shown to have resistance to metals [79], with some strains able to produce organic acids, IAA, siderophores, and solubilize phosphate [80] as found for *Tsukamurella* sp. N1171 here. In addition, it was described that *Tsukamurella spumae* (a species phylogenetically related to our isolate) produces surfactant compounds, which improve the use of hydrophobic compounds as carbon and energy sources [81].

Therefore, based on the prospection of the current study, bacteria strains obtained from the *B. linearis* root zone can be considered as potential biotechnological tools for phytoremediation programs in Cu mine tailings. The ACC activity was the main PGP trait found in isolated bacteria. This PGP trait has been considered a key PGP mechanism to ameliorate abiotic stress in plants [82]. Additionally, several studies have reported positive effects of ACC-producing bacteria activity on the fresh and dry weight of seedlings, also reducing the ethylene production under drought stress [83]. On the other hand, some bacteria strains showed the biological N-fixation and phosphate solubilization traits, which are key processes for plants growing in mine tailings considering their low N and P contents. The microbial communities from the root zone showed the highest acid phosphatase and urease activity. Additionally, certain strains were able to produce IAA and siderophores, which is the most abundant type of auxin and can stimulate cellular elongation, vascular tissue differentiation, growth, apical dominance, and lateral root initiation [84]. All bacteria strains (except for *Frondihabitans* sp. GRS42) were able to produce siderophores, which improve the Fe acquisition under Fe-limited conditions [85]. Also, siderophore-producing bacteria have been shown to improve the chlorophyll content and growth of several plants in metal contaminated soils [86]. Therefore, we propose the evaluation of a synthetic microbial community based on the bacteria strain obtained here for promoting revegetation since the interaction among different organisms may play a key role in the survival of the species living under abiotic stress [87].

## 5. Conclusions

Bacterial strains isolated from the root zone of *B. linearis* showed PGP traits associated with nutrient acquisition, plant development, and metal(loid) resistance, among others. This suggests that bacteria derived from the root zone of *B. linearis* have the potential to aid in the establishment and growth of the plant, even in the challenging conditions commonly found in mine tailings. This represents a potential biotechnological tool for further ecological management of mine tailings. Nevertheless, further PGP analyses are essential to develop a consortium or synthetic community comprising bacterial strains identified in this study. Additionally, the obtained bacterial strains might possess other advantageous attributes beneficial for plants, such as biosurfactant production and alleviation of water-related stress. These traits could prove valuable for the phytoremediation of mine tailings. Hence, we recommend exploring the bioaugmentation effects of these beneficial native microbial taxa, which have adapted to the conditions of mine tailings and that might contribute to support the growth and development of native plant species.

## Figures and Tables

**Table 1:** Physicochemical properties of bulk soil and root-zone samples (*n* = 5; mean ± s.e.).

Properties	Bulk Soil				Root Zone			
pH	4.77	±	0.13	a	5.30	±	0.23	a
CE (S cm^−1^)	820.1	±	286.3	a	559.6	±	349.2	a
TOC (%)	0.64	±	0.13	a	1.11	±	0.24	a
P_Total_	0.20	±	0.00	a	0.20	±	0.09	a
P_Available_	16.09	±	0.92	a	12.86	±	1.82	a
N_Total_	0.06	±	0.02	a	0.08	±	0.03	a
K_Total_	1.14	±	0.04	a	1.15	±	0.05	a
Ca_Total_	2.50	±	0.03	a	2.35	±	0.08	a
Mg_Total_	0.87	±	0.02	a	0.81	±	0.04	a
S_Total_	0.65	±	0.10	a	0.52	±	0.05	a
Fe_Total_	5.06	±	0.06	a	4.81	±	0.08	b
Cu_Total_	977.04	±	137.02	a	510.31	±	39.88	b
Zn_Total_	51.48	±	0.78	a	48.03	±	1.52	a
As_Total_	23.77	±	1.99	a	20.80	±	1.20	a
Mn_Total_	994.23	±	36.94	a	980.00	±	46.79	a
Mo_Total_	37.11	±	2.39	a	41.80	±	3.42	a
Cu_H2O_	16.70	±	6.20	a	1.40	±	0.34	b
Zn_H2O_	1.14	±	0.31	a	0.29	±	0.14	b
Ca_H2O_	58.21	±	19.55	a	41.73	±	17.07	a
Mg_H2O_	37.98	±	13.08	a	23.78	±	10.25	a

Different letters (‘a’ and ‘b’) indicate significant differences (*p* ≤ 0.05) according to the *t*-test. Total nutrient and metal(loid) contents are expressed in mg kg^−1^. Available contents (H_2_O) are expressed in mg L^−1^. CE = electrical conductivity; TOC = total organic carbon.

**Table 2:** Plant growth-promoting traits of bacterial isolates from the root zone of *Baccharis linearis*.

Bacterial Strain	NF	PS	ACC	IAA	SID
*Pseudomonas* sp. YL167	+	+	+	+	+
*Arthrobacter* sp. pp5	+	+	+	+	+
*Frondihabitans* sp. GRS42	−	+	+	−	−
*Acinetobacter* sp. 47	−	−	+	−	+
*Tsukamurella* sp. N1171	+	+	+	+	+

NF = growth in N-free culture medium; PS = phosphate solubilization; ACC = 1-aminocyclopropane-1-carboxylate deaminase activity; IAA = indole acetic acid production; SID = production of siderophores. The bacterial strains with positive plant growth-promoting traits are indicated by “+”, while the absence of such traits is shown by “−”.

## Data Availability

The data supporting the reported results in the present study are available on request from the corresponding author or the first author.
